# Psychometric evaluation of the Kessler 10 measure of psychosocial distress using the 2022 South African population-based household survey

**DOI:** 10.1007/s12144-026-09563-y

**Published:** 2026-05-30

**Authors:** Sbonelo Chamane, Musawenkosi Mabaso, Ronel Sewpaul, Lehlogonolo Makola, Sean Jooste, Sizulu Moyo, Nobuhle Mchunu, Yusentha Balakrishna, Nompumelelo Zungu, Olive Shisana, Khangelani Zuma

**Affiliations:** 1 Public Health, Societies and Belonging, Human Sciences Research Council, Pretoria, South Africa; 2 School of Public Health, Faculty of Health Sciences, University of the Witwatersrand, Johannesburg, South Africa; 3 School of Public Health and Family Medicine, University of Cape Town, Cape Town, South Africa; 4 Biostatistics Research Unit, South African Medical Research Council, Durban, South Africa; 5 School of Nursing and Public Health, University of KwaZulu-Natal, Durban, South Africa; 6 Department of Psychiatry and Mental Health, University of Cape Town, Cape Town, South Africa; 7 Evidence Based Solutions Consulting Pty Ltd, Cape Town, South Africa

**Keywords:** Psychological distress, IRT, Psychometric validation, DIF, South Africa

## Abstract

The Kessler Psychological Distress Scale (K10) is widely used in epidemiological research but requires validation within South Africa’s multilingual setting. This study evaluated the psychometric properties of the K10 using national population-based survey data and Item Response Theory (IRT). Graded Response Models (GRM) were used to assess factor loadings, item discrimination, and difficulty parameters, while Test Information Curves evaluated measurement precision across the distress continuum. Differential Item Functioning (DIF) analysis examined language effects, supporting cross-group reliability. All items exhibited high factor loadings (0.86–0.96) and substantial communalities (> 0.74) in the unidimensional GRM. Although the multidimensional GRM showed marginal improvement, relatively low factor loadings limited its suitability. All items had high discrimination parameters, ranging from 2.89 (K1) to 5.78 (K6). Difficulty parameters (β1–β4) followed the expected increasing pattern across response categories. Items K6 and K9 were the most informative given by the highest discrimination values. Internal consistency was excellent (Cronbach’s alpha and McDonald’s Omega total = 0.95), while marginal reliability under the unidimensional IRT model was moderate (0.65). Items 4, 5, 7, 8, and 10 were flagged for possible DIF by English-speaking status; effect sizes were negligible (R² < 0.002). This study provides robust evidence supporting the psychometric soundness of the K10 in a South African context. The scale functions as a unidimensional, internally consistent, and discriminating measure of psychological distress. Marginal reliability and information curves indicate it is most precise for detecting moderate-to-high distress levels, with potential for refinement in screening for milder symptoms.

## Introduction

Despite evidence of an increasing burden of mental health disorders globally (World Health Organization, 2022), nationally representative data on the prevalence of mental health disorders in South Africa are limited. For many years, the primary source of data on mental health was the South African Stress and Health Survey (SASH), along with data from smaller studies focused on specific population groups or from treatment centers (Breedt et al., 2023; Herman et al., 2009; Peltzer et al., 2012). A survey conducted by the University of Witwatersrand across nine provinces provides the most recent data using a nationally representative sample. This study found that at a national level, 25.7% reported probable depression, 17.8% probable anxiety, and 23.6% high adverse childhood experiences exposure (Uddin et al., 2018; Craig et al., 2022).

Since 2002, the Human Sciences Research Council (HSRC) in South Africa has carried out six nationally representative surveys to estimate the burden of HIV infection in the country (Shisana et al., 2005, 2009, 2014; Shisana & Simbayi, 2002; Simbayi et al., 2021; Zuma et al., 2022). The inaugural survey was conducted with support from the Nelson Mandela Foundation and was a landmark effort that provided essential, trustworthy data on the country’s HIV prevalence, incidence, and associated behavioural patterns in South Africa. Over the years, this survey has evolved to include a measure for mental health to address the absence of mental health of national-level estimates (Shisana et al., 2014; Simbayi et al., 2021; Zuma et al., 2025).

The Kessler Psychological Distress Scale (Kessler 10 or K10) is a widely used measure of psychological distress, developed in 1992, and was incorporated into the South African National HIV Prevalence, Incidence and Behaviour Survey (SABSSM) in 2012 (Kessler & Mroczek, 1992; Shisana et al., 2014). The scale is a ten-item measure utilised for screening non-specific psychological distress in the general population (Kessler et al., 2002, 2003). Since then, the K10 has been used in the SABSSM surveys to assess mental health status alongside HIV-related data, providing insights into the prevalence of psychological distress, and specifically investigate the relationship between psychological distress and HIV infection in South Africa (Craig et al., 2022).

The K10 has gained growing recognition in epidemiological research and is now among the most widely-used self-reported measures of psychological distress. (Kessler et al., 2002, 2003). In the last two decades, the K10 has demonstrated strong psychometric properties that provide an ability to distinguish disorder cases and non-cases as categorised in the Diagnostic and Statistical Manual of Mental Disorders, Fourth Edition (DSM-IV). Studies have validated the K10 and its shorter version K6, against recognized diagnostic interviews such as the World Health Organisation (WHO) Composite International Diagnostic Interview (CIDI) and the Structured Clinical Interview for DSM-IV (SCID) (Furukawa et al., 2003; Kessler et al., 2010). Evidence from earlier studies conducted in Europe suggested that both the K10 and K6 scales have reliability and validity in epidemiological studies, effectively screening for mood and anxiety disorders with good sensitivity (Lehmann et al., 2023).

Recent research conducted worldwide further supports the reliability and validity of the K10 scales in diverse populations, including cross-cultural settings and low- and middle-income countries. Validation studies have confirmed the utility of translated versions of the K10 (Easton et al., 2017; Kislitsyn et al., 2025). Further studies have reinforced the scales’ applicability for screening psychological distress and serious mental illness across different clinical and community populations, with area under the curve (AUC) values reflecting good discriminative ability and a good consistency (Andersen et al., 2011; Lehmann et al., 2023. This growing body of evidence affirms that the K10 is an effective screening instrument with strong psychometric support in both high-income and developing country contexts (Hoffman et al., 2022). Despite its wider popularity, this scale has some key limitations, such as the lack of specificity, the lack of diagnostic capabilities, and reduced validity when used with certain populations or in specific cultural contexts (Ametaj et al., 2024; Pereira et al., 2019).

In South Africa, several studies have examined the psychometric properties of the K10 scale, focusing on its reliability, validity, and factor structure. A large outpatient study, consisting of 2,591 participants, reported good internal consistency between the items of the scale (Cronbach’s alpha = 0.84; Omega total = 0.88) and supported a primarily unidimensional factor structure, although exploratory analyses suggested a four-factor model that appeared less stable (Hoffman et al., 2022). The authors concluded that the K10 is a suitable tool for the South African context but emphasized the necessity of establishing culturally relevant cut-off scores to optimize its clinical utility. The South African Stress and Health Study evaluated the K10 and its shorter version, the K6, against clinical diagnostic interviews using a nationally representative sample, and showed that the K10 showed moderate ability to discriminate cases of depression and anxiety, with areas under the Receiver Operating Characteristic (ROC) curve of 0.73 and 0.72, respectively (Andersen et al., 2011). However, positive predictive value was low at 23%, meaning that less than one in four people who screened positive actually had the condition. This reinforces that the K10 cannot be relied on as a standalone diagnostic instrument in this population. Notably, the study found reduced discriminative accuracy among Black South Africans compared to other groups (Andersen et al., 2011). This highlights the need for further validation across diverse populations and for examining the underlying psychometric structure, which could help explain the limited diagnostic accuracy and inform cultural and clinical adaptations of the scale in contexts such as South Africa.

Recent research has underscored the importance of cultural and linguistic adaptation, recommending the development of population-specific cut-off points and consideration of local expressions of distress. Cross-cultural investigations across several African countries reinforce the importance of such local validation efforts before clinical or epidemiological application (Andersen et al., 2011; Hoffman et al., 2022). Although some studies have demonstrated strong psychometric performance of the K10 in several high-income countries, little academic literature exists on its validity in African populations using advanced psychometric methods (Ametaj et al., 2024). The psychometric properties of a measurement instrument, such as the K10, are often evaluated using the Classical Test Theory (CTT) or the Item Response Theory (IRT) (DeMars, 2018; Rezapour et al., 2021). The CTT uses reliability indices like Cronbach’s alpha to assess internal consistency, assumes uniform error variance across all respondents and evaluates test scores based on the total sum of item scores (Ayanwale et al., 2022; DeMars, 2018). Conversely, IRT provides more detailed insights through tools like the Test Information Function (TIF), which reflects the precision of measurement across different levels of the latent trait, treats measurement tools as inherently imperfect, allowing for variation in error depending on a respondent’s trait level and the probability of a specific response is modelled based on the person’s position on the latent trait continuum and the item’s characteristics (Idaka & Idaka, 2014).

There is very limited research in South Africa on the application of IRT models to assess the item-level performance and measurement precision of the K10. This paper aims to examine the distribution and psychometric performance of the 10-item Kessler Psychological Distress Scale (K10) in a nationally representative sample of South African adults by exploring its dimensional structure, evaluating item-level properties and whether the translated versions of the K10 function equivalently across language groups in South Africa.

## Methodology

### Study data and population

The sixth SABSSM Survey was a cross-sectional, population-based household survey conducted in 2022 to estimate the burden of HIV infection (Zuma et al., 2025). For sampling, a two-stage stratified cluster sampling design was used where the primary sampling unit consisted of 1,000 small area layers (SALs) stratified by province, locality type, and race group. An additional 1,114 SALs were included to allow for district-level oversampling. Each SAL targeted 15 Visiting Points (VPs), yielding 32,160 in total with only 27,005 (91.7%) valid VPs. Of the valid VPs 21,615 agreed to participate, resulting in a household response rate of 80%. The second sampling unit was all individuals residing in the selected households. The survey population excluded individuals residing in institutions such as old-age homes, orphanages, homeless persons living on the streets or in shelters, student residences, and uniformed-service barracks. Additionally, individuals who were unable to provide informed consent based on their understanding and comprehension of the consent process and information provided were also excluded. Data were collected electronically using tablets that contained a household questionnaire and individual behavioural questionnaires tailored for parents or guardians of children aged 0–11 years, children aged 12–14 years, and individuals aged 15 years and older. This study focuses on the questionnaire for individuals aged 15 years and older.

### Ethical clearance

Ethical approval for the survey was granted by the Human Sciences Research Council (HSRC) Research Ethics Committee (REC) with protocol approval number REC: 4/18/11/15. Approval was also granted by the Associate Director for Science, Center for Global Health (CGH), Centers for Disease Control and Prevention (CDC). The study was conducted in accordance with the relevant institutional and ethical guidelines.

### Characteristic traits

Individual sex (female, male), age group (15–24, 25–34, 35–44, 45–54, 55–64, 65+), race group (Black African, Other races), highest education obtained (non/primary school, secondary school, tertiary education), employment status (employed, unemployed), locality type (urban areas, rural informal [tribal areas], rural formal [farm areas]). These variables were presented solely to describe the sample characteristics and were not included as covariates or used to adjust the model.

### Assessment of differential item functioning (DIF)

For DIF analysis, the language spoken most often at home was used as the variable. Respondents reported their main language as one of the following official South African languages: Afrikaans, English, IsiNdebele, SiSwati, IsiXhosa, IsiZulu, Sesotho, Sepedi, Setswana, Tshivenda, or Xitsonga. A binary variable was generated with English coded as one level, since the original Kessler Psychological Distress Scale was developed in English, and all other ten languages grouped as the second level. The questionnaire was translated into each of the official languages to ensure accessibility.

### Items

The study used the K10 to assess symptoms of non-specific psychological distress over the previous 30 days, designed to specifically capture experiences related to anxiety and depression. Respondents were asked how often they experienced each symptom using a five-point Likert-type scale, where 1 = None of the time, 2 = A little of the time (rarely), 3 = Some of the time, 4 = Most of the time, and 5 = All of the time. These items are as follows:

- K1 During the last 30 days, about how often did you feel tired out for no good reason?
- K2 During the last 30 days, about how often did you feel nervous?
- K3 About how often did you feel so nervous that nothing could calm you down?
- K4 About how often did you feel hopeless?
- K5 During the last 30 days, about how often did you feel restless or fidgety?
- K6 About how often did you feel so restless you could not sit still?
- K7 About how often did you feel depressed?
- K8 During the last 30 days, about how often did you feel that everything was an effort?
- K9 About how often did you feel so sad that nothing could cheer you up?
- K10 About how often did you feel worthless?

### Data analyses

Descriptive statistics were used to summarise the responses per item using proportions and percentages. The K10 was analysed using Item Response Theory (IRT), specifically through the Graded Response Model (GRM) for ordinal response data. Both unidimensional and multidimensional GRMs were fitted using the *mirt* package in R version 4.4.2 (2024-10-31). The unidimensional model assumed that all items were measured as a single underlying latent trait, in this case psychosocial distress. The multidimensional model allowed for the items to reflect as multiple related but distinct traits. To assess the structure and explanatory power of the models, both factor loadings and communalities were examined. The two GRM models were compared using Akaike Information Criterion (AIC), Sample-Size Adjusted BIC (SABIC), Bayesian Information Criterion (BIC), Log-Likelihood (logLik), and Likelihood Ratio Test (X²), where a lower AIC, SABIC, and BIC and higher logLik, coupled with a significant X² indicated model improvement. The Item Discrimination and Difficulty parameters were presented to evaluate the performance of individual K10 items for the selected model. Model fit was evaluated using the M2 limited-information statistic, from which approximate fit indices including Root Mean Square Error of Approximation (RMSEA), Comparative Fit Index (CFI), Tucker-Lewis Index (TLI), and the Standardised Root Mean Square Residual (SRMR) were derived based on the discrepancy between observed and model-implied response patterns. A good model fit is typically indicated by RMSEA values below 0.06, CFI and TLI values of 0.95 or higher, and SRMR values below 0.08. RMSEA values between 0.06 and 0.08 may still reflect acceptable fit. These indices were used to assess the fit of the unidimensional K10 model. Importantly, the factor scores were derived using IRT, and the reported fit statistics (RMSEA, CFI, TLI, and SRMR) are based on IRT estimation rather than traditional factor analysis. The Test Information Function (TIF) were used to assess the precision on the latent trait. The Cronbach’s alpha and McDonalds Omega were used to evaluate internal consistency. While McDonald’s Omega total is often presented as a more accurate approximation of a scale’s reliability than the Cronbach’s alpha, both indices are often used as indicators of scale structure (Peters, 2014). The empirical marginal reliability was used to evaluate reliability of the estimated latent trait scores. Differential item function was assessed using the *lordif* package in R v4.4.2. The analysis was stratified by language, with English speakers compared against a grouped category representing the other ten official South African languages. Items identified as showing potential DIF were examined further. For each flagged item, three DIF statistics were evaluated: the non-uniform DIF test (Pr(χ²_12_,1), R²_12_, and Δβ1), the uniform DIF test (Pr(χ²_13_,2) and R²_13_), and the overall DIF test (Pr(χ²_23_,1) and R²_23_). The McFadden pseudo-R² (R²) values were used to assess the magnitude of DIF, with thresholds of R² ≥ 0.02 generally considered indicative of practically meaningful DIF. All the analyses were weighted to account for the complex multilevel unequal sampling probabilities.

in the survey design.

## Results

The study included questionnaire responses from 47,609 participants aged 15 years and older who responded to at least one of the 10 Kessler items. Almost half were 15–34 years old (47.2%), and 53.6% were female. The majority of participants were Black African (79.8%), had completed secondary schooling (62.8%), were unemployed (66.3%), lived in urban areas (67.8%) and the language most spoken at home was not English (91.8%, Table 1.)

**Table 1:** Sociodemographic characteristics of participants who responded to the Kessler scale

Variables	*N*	%
Total	47,609	100
Sex of the respondent		
Male	19,644	46.4
Female	27,943	53.6
Age groups (years)		
15–24	11,850	22.6
25–34	10,418	24.6
35–44	8,516	20.6
45–54	6,443	13.4
55–64	5,459	10.1
65+	4,923	8.7
Race groups		
Black African	41,957	79.8
Other races	5,587	20.2
Highest education obtained		
Non/Primary school	10,765	17.8
Secondary school	29,592	62.8
Tertiary education	6,814	19.4
Employment status		
Employed	13,558	33.7
Unemployed	33,032	66.3
Locality type		
Urban areas	29,832	67.8
Rural informal (tribal areas)	12,571	26.1
Rural formal (farm areas)	5,206	6.1
Language most spoken at home		
Non-English*	45,238	91.8
English speaker	2,371	8.2

^*^Afrikaans, IsiNdebele, SiSwati, IsiXhosa, IsiZulu, Sesotho, Sepedi, Setswana, Tshivenda, or Xitsonga

Table 2 presents the distribution of responses across the K10 in the nationally representative South African sample. For each item, the majority of respondents selected “None of the time,” ranging from 76.6% for tiredness (K1) to 86.6% for restlessness to the point of not being able to sit still (K6). Across items, responses indicating high frequency of distress (Most of the time and all of the time) were relatively low, typically below 3%.

**Table 2:** Distribution of Kessler K10 item responses

Item	Question	None of the time	A little of the time (rarely)	Some of the time	Most of the time	All of the time
K1	During the last 30 days, about how often did you feel tired out for no good reason?	36,526 (76.6%)	5,109 (10.7%)	4,267 (9.0%)	1,267 (2.7%)	499 (1.0%)
K2	During the last 30 days, about how often did you feel nervous?	39,712 (83.3%)	3,863 (8.1%)	2,963 (6.2%)	798 (1.7%)	332 (0.7%)
K3	About how often did you feel so nervous that nothing could calm you down?	40,892 (85.8%)	3,265 (6.8%)	2,500 (5.2%)	736 (1.5%)	275 (0.6%)
K4	About how often did you feel hopeless?	38,661 (81.1%)	4,064 (8.5%)	3,386 (7.1%)	1,154 (2.4%)	403 (0.8%)
K5	During the last 30 days, about how often did you feel restless or fidgety?	40,747 (85.5%)	3,213 (6.7%)	2,609 (5.5%)	798 (1.7%)	301 (0.6%)
K6	About how often did you feel so restless you could not sit still?	41,303 (86.6%)	2,883 (6.0%)	2,460 (5.2%)	744 (1.6%)	278 (0.6%)
K7	About how often did you feel depressed?	38,612 (81.0%)	3,992 (8.4%)	3,579 (7.5%)	1,099 (2.3%)	386 (0.8%)
K8	During the last 30 days, about how often did you feel that everything was an effort?	39,672 (83.2%)	3,137 (6.6%)	3,144 (6.6%)	1,151 (2.4%)	564 (1.2%)
K9	About how often did you feel so sad that nothing could cheer you up?	40,590 (85.2%)	3,102 (6.5%)	2,768 (5.8%)	881 (1.8%)	327 (0.7%)
K10	About how often did you feel worthless?	40,842 (85.7%)	2,867 (6.0%)	2,650 (5.6%)	900 (1.9%)	409 (0.9%)

All items demonstrated high factor loadings in the unidimensional model (range: 0.86 to 0.96; Table 3), with corresponding communalities above 0.74. This indicates that each item shares a substantial proportion of its variance with the underlying latent factor of psychological distress. The sum of squared loadings for the unidimensional model was 8.76, suggesting that the single factor accounted for approximately 87.6% of the common variance across all items.

**Table 3:** Factor loadings, communalities and model fit indices for unidimensional and multidimensional graded response models (K10)

Item	Unidimensional		Multidimensional		
	Factor Loading (F1)	Communality (h²)	Factor Loading (F1)	Factor Loading (F2)	Communality (h²)
K1	0.86	0.74	0.76	0.26	0.76
K2	0.92	0.85	0.79	0.38	0.94
K3	0.93	0.87	0.86	0.21	0.88
K4	0.94	0.89	0.93	−0.01	0.87
K5	0.95	0.90	0.93	0.03	0.88
K6	0.96	0.92	0.95	−0.01	0.90
K7	0.93	0.86	0.93	−0.07	0.83
K8	0.95	0.90	0.97	−0.11	0.88
K9	0.96	0.92	0.98	−0.10	0.91
K10	0.96	0.92	0.99	−0.15	0.92
Total (SS Loadings)	8.76		8.31	0.30	
Model Fit Indices	**Unidimensional**		**Multidimensional**		
Akaike Information Criterion (AIC)	327,496,950.00		324,587,454.00		
Sample-Size Adjusted BIC (SABIC)	327,497,570.00		324,588,186.00		
Bayesian Information Criterion (BIC)	327,497,729.00		324,588,373.00		
Log-Likelihood (logLik)	−163,748,425.00		− 162,293,668.00		
Likelihood Ratio Test (X²)			2,909,513.93		
Degrees of Freedom (df)			9		
p-value			< 0.001		

In the multidimensional model, most items continued to load strongly on the first factor (F1), while loadings on the second factor (F2) were low, ranging from − 0.15 to 0.38. The variance explained by the first factor in the multidimensional model (SS loading = 8.31) was slightly lower than in the unidimensional model, and the second factor contributed only marginally (SS loading = 0.30). These findings support the appropriateness of a unidimensional structure for the K10, with little evidence that a second factor adds meaningful explanatory value.

Fit statistics comparing the unidimensional and multidimensional graded response models are shown in Table 3. Although the likelihood ratio test was significant (χ² = 2,909,513.93, df = 9, *p* < 0.001) and the AIC and SABIC dropped by over 2.9 million points, suggesting a statistical improvement with the multidimensional model, the second factor loadings were minimal and the gain in interpretability was negligible. This supports the continued use of a unidimensional structure for the K10. The unidimensional model demonstrated acceptable fit indices: RMSEA = 0.058 (90% CI: 0.055–0.062), SRMSR = 0.072, TLI = 0.99, and CFI = 0.997, supporting the use of a unidimensional model for further analysis. These findings affirm the K10’s structural validity as a measure of a single latent dimension of psychological distress.

Table 4 summarises the item discrimination (α) and difficulty (β) parameters estimated under the unidimensional graded response model. Discrimination parameters ranged from 2.89 (K1) to 5.78 (K6), indicating that all items are highly informative in distinguishing between different levels of psychological distress. Difficulty parameters (β1–β4) followed an expected increasing pattern across response categories, suggesting proper functioning of the ordinal scale and appropriate ordering of response options. The Cronbach’s alpha was very high (0.95) and Omega total was 0.95, indicating strong internal consistency among the scale items. However, the marginal reliability under the unidimensional IRT model was 0.65, indicating that the scale provides reliable measurement primarily within a narrower band of psychological distress.

**Table 4:** Item discrimination and difficulty parameters for the unidimensional graded response model

Item	α	β_1_	β_2_	β_3_	β_4_
K1	2.89	0.98	1.45	2.15	2.69
K2	4.01	1.14	1.52	2.12	2.60
K3	4.46	1.26	1.60	2.13	2.68
K4	4.88	1.07	1.45	1.97	2.46
K5	5.24	1.19	1.53	2.02	2.48
K6	5.78	1.27	1.58	2.03	2.53
K7	4.20	1.07	1.44	1.98	2.50
K8	4.97	1.17	1.49	1.94	2.35
K9	5.62	1.22	1.53	1.98	2.46
K10	5.57	1.23	1.55	1.98	2.41

**Fig. 1:**
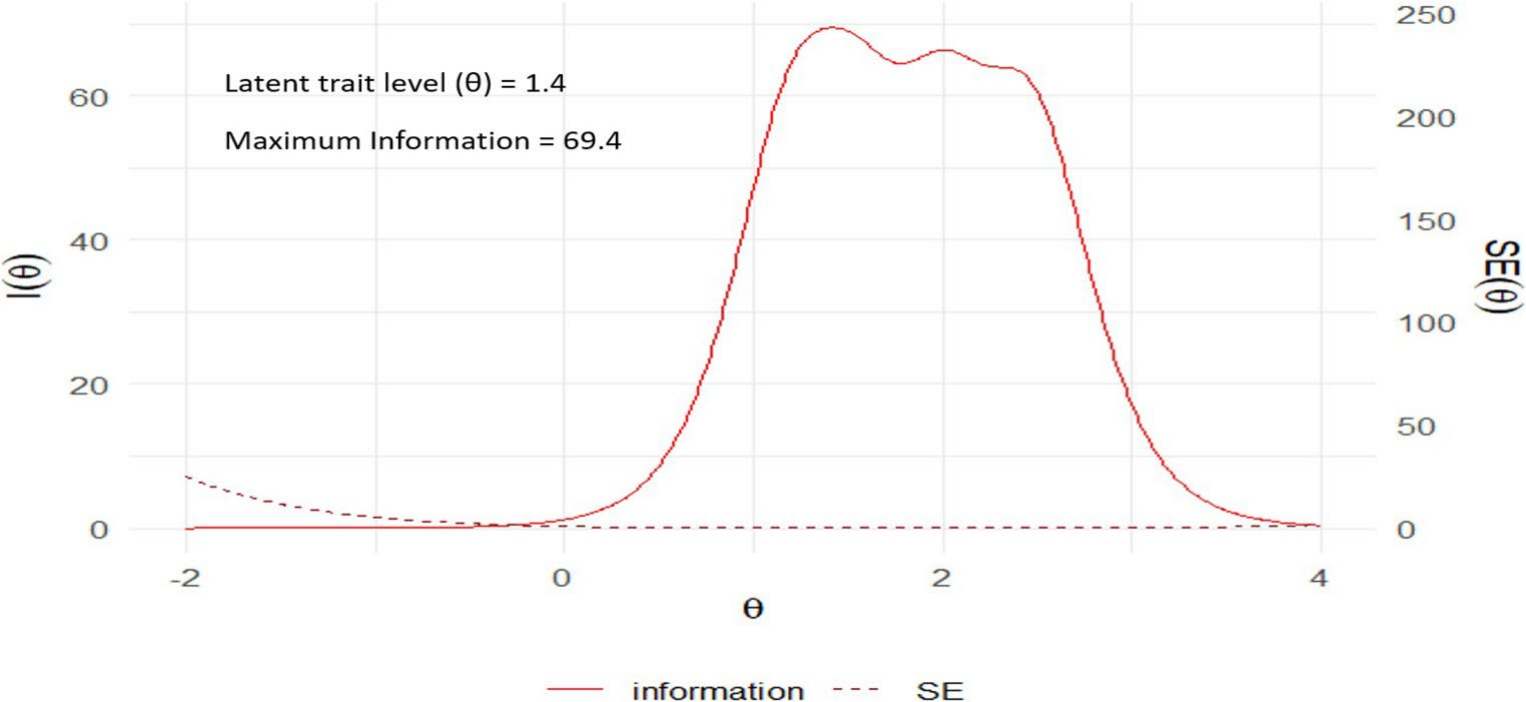
Test information curves for the K10 scale

Figure 1 presents the Test Information Function (TIF) for the K10 scale based on the unidimensional Graded Response Model. The TIF (left panel) indicates that the K10 scale provides maximum measurement precision at a latent trait level (θ) of approximately 1.4, with a peak information value of 69.4. This suggests that the scale is particularly sensitive for detecting moderate to high levels of psychological distress.

**Fig. 2:**
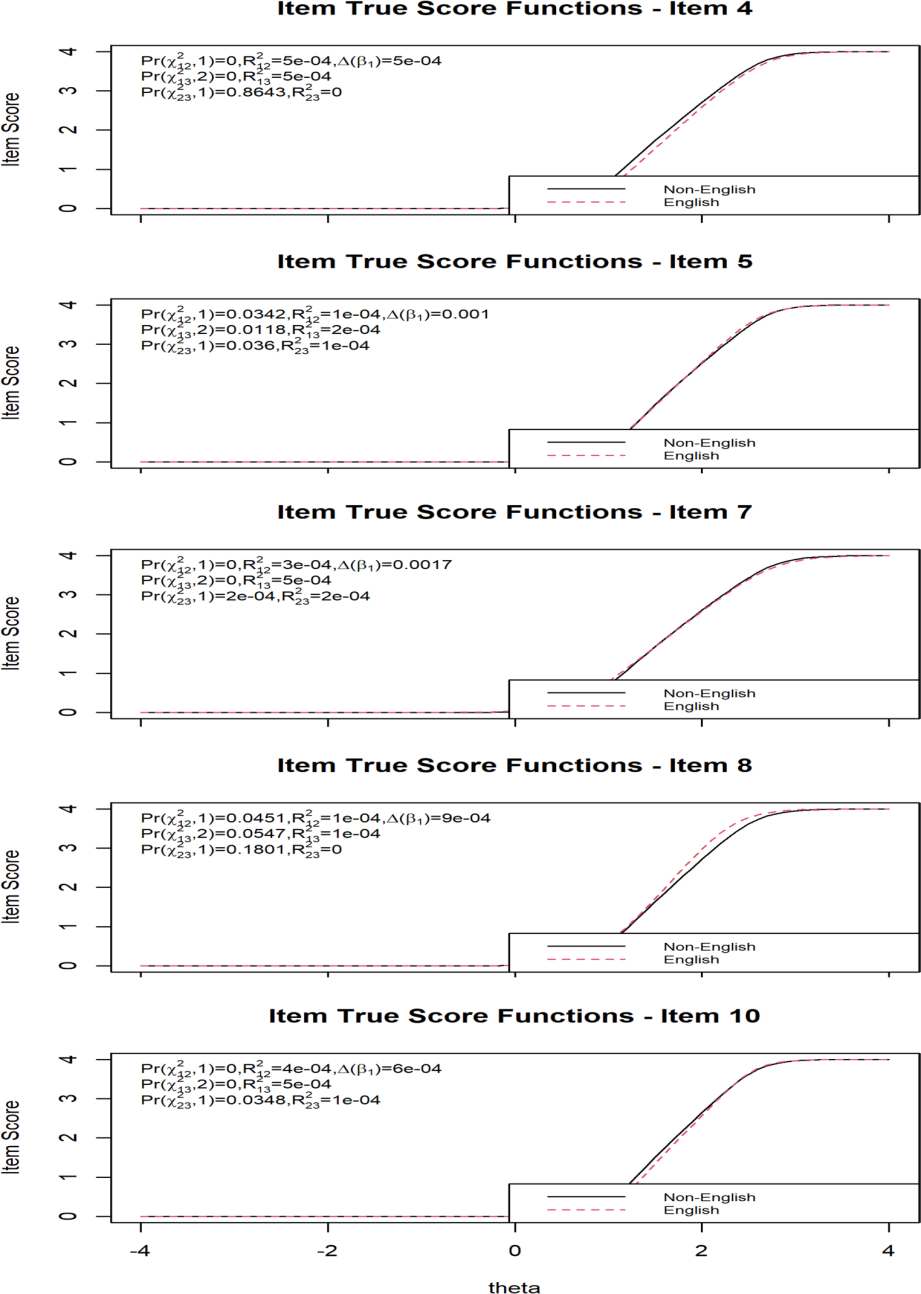
Item true score functions for K10 items with suspected DIF by English-speaking status

Figure 2 shows that Items 4, 5, 7, 8, and 10 of the K10 were flagged for possible DIF by English-speaking status. For Item 4, the non-uniform DIF test was Pr(χ²_12_,1) < 0.001 with R²_12_=0.0005 and Δβ1 = 0.0005, the uniform DIF test was Pr(χ²_13_,2) = < 0.001 with R²_13_=0.0005, and the overall DIF test was Pr(χ²_23_,1) = 0.8643 with R²_23_=0, indicating no evidence of DIF. Item 5 showed Pr(χ²_12_,1) = 0.0342 with R²_12_=0.0001 and Δβ1 = 0.001, Pr(χ²_13_,2) = 0.0118 with R²_13_=0.0002, and Pr(χ²_23_,1) = 0.036 with R²_23_=0.0001, suggesting statistically significant uniform and non-uniform DIF but with negligible effect sizes. Item 7 produced Pr(χ²_12_,1) < 0.001 with R²_12_=0.0003 and Δβ1 = 0.0017, Pr(χ²_13_,2) = < 0.001 with R²_13_=0.0005, and Pr(χ²_23_,1) = < 0.001 with R²_23_=0.0002, again statistically significant but trivial in magnitude. For Item 8, results were Pr(χ²_12_,1) = 0.0451 with R²_12_=0.0001 and Δβ1 = 0.0009, Pr(χ²_13_,2) = 0.0547 with R²_13_=0.0001, and Pr(χ²_23_,1) = 0.1801 with R²_23_=0, which showed marginal statistical significance but no meaningful effect size. Finally, Item 10 showed Pr(χ²_12_,1) < 0.001 with R²_12_=0.0004 and Δβ1 = 0.0006, Pr(χ²_13_,2) < 0.001 with R²_13_=0.0005, and Pr(χ²_23_,1) = 0.0348 with R²_23_=0.0001, indicating statistically significant DIF but again with a negligible effect. Across all items, while some chi-square tests reached statistical significance, the effect size estimates (all R² values < 0.002) suggest that none of the flagged items displayed practically meaningful DIF.

## Discussion

This study aimed to examine the distribution and psychometric properties of the 10-item Kessler Psychological Distress Scale (K10) in a nationally representative sample of South African adults by exploring its dimensional structure, evaluating item-level properties (factor loadings, discrimination, and difficulty parameters), and assessing the scale’s internal consistency, reliability and information function. Across items, responses indicating high frequency of distress (Most of the time and all of the time) were relatively low. Despite this, South Africa ranks among the lowest globally in mental well-being, with 35% of South African respondents classified as either distressed or struggling in 2023 (Sapien labs, 2024). This contrast between item-level response patterns and broader population mental health underscores the complexity of distress reporting, possibly influenced by stigma, cultural norms around emotional expression, or the episodic nature of psychological symptoms.

The structural analysis supported a unidimensional model; its unidimensional structure justified the use of the IRT (Ip, 2010; Reise et al., 2025). Additionally, it aligns with the original intent of the K10 as a single-factor tool, justifying the use of a total score for assessing general psychological distress in this population (Sampasa-Kanyinga et al., 2018). Although some prior research has reported multidimensional structures in certain —often separating anxiety and depressive symptoms (Pereira et al., 2019), the study findings suggest that such distinctions may be less critical in population-level screening where brevity and efficiency are paramount. Sunderland suggest that the use and interpretation of the scale depend on the population and purpose of assessment, where, similar to our findings, screening was found to be more suitable for detecting general psychological distress in the population, whereas in clinical populations, distinguishing anxiety and depression becomes more important (Sunderland et al., 2012). Although our findings validate the use of the scale for population-level screening, its performance in South African clinical settings was previously found to fall short of acceptability (Andersen et al., 2011), highlighting the need for further studies to establish its clinical validity. Nonetheless, cross-cultural norms and stigma influences need to be considered, as they may affect how individuals interpret and respond to items, potentially impacting the scale’s accuracy across different groups.

As observed on the study by Brooks (Brooks et al., 2006), all the K10 items showed a high discrimination, highlighting their effectiveness in differentiating between individuals with varying levels of distress. Contrary to our finding, a study conducted in Ecuadorians among adults and adolescents showed that 9 of the 10 items had adequate discrimination and difficulty, with item 8 showing poor factor loading, weaker discrimination and problematic difficulty levels reinforcing our earlier suggestion of the importance of considering the cross cultural and contextual factors in considering the performance of the items of the scale (Larzabal-Fernandez et al., 2024). Nonetheless, our finding reinforces the validity of the scale particularly for public health surveillance, where identifying those at greatest risk is essential. However, the item information functions revealed that the scale is most informative at moderate to high levels of distress. This suggests that while the K10 is suitable for identifying individuals with clinically significant symptoms, it may be less sensitive for detecting mild or subclinical distress, potentially limiting its utility in early intervention contexts.

The findings revealed that the K10 demonstrated high internal consistency while it provided a low marginal reliability. The finding on internal consistency aligns with prior research, which has also reported high reliability Cronbach’s α = 0.88 (Sampasa-Kanyinga et al., 2018) and Cronbach’s α = 0.93 (Fassaert et al., 2009). A Brazilian study on the Cross-cultural adaptation and psychometric properties showed a K10 high reliability score at 0.87 (Peixoto et al., 2021). Similar to our context, previous research in South Africa reported a high internal consistency of the K10 with a Cronbach’s alpha of above 0.80 (Hoffman et al., 2022). However, limited research is available on the marginal reliability within this population, which is a suitable test of reliability for IRT (Brown, 2017). These findings suggest that while the K10 is a reliable screening tool at the aggregate level, caution should be exercised in using it to make inferences based on single-item scores. The low marginal reliability potentially reflects issues such as overlapping content among items, ceiling or floor effects, or variability in how respondents interpret individual questions.

This analysis indicated that English-speaking and non-English–speaking respondents showed statistically significant differential item functioning (DIF) for Items 4, 5, 7, 8, and 10 of the K10; however, the effect sizes were negligible (all R² < 0.002), indicating no practically meaningful bias. This suggests that while certain items appeared to function differently between language groups, the magnitude of difference was too small to compromise the scale’s validity. This finding aligns with existing cross-linguistic validation studies (Easton et al., 2017; Kislitsyn et al., 2025). For the South African context, where the K10 has been translated into multiple local languages, these findings support the continued use of the instrument, as the flagged items do not meaningfully compromise the validity of comparisons between English and non-English speakers.

### Limitations

The K10 uses self-reported items, which may be subject to social desirability bias and recall bias. While the graded response model was appropriate for the polytomous structure of the K10, model fit and parameter estimates could be affected by unmodeled multidimensionality or local dependence among items, which future studies should explore further. A key limitation of this analysis is the assumption that participants completed the questionnaire in the language they speak at home, which may not always reflect their preferred or most comfortable language for completing a research questionnaire. The grouping approach used for the DIF analysis compared English versus a pooled “all other languages” group; this approach may have masked important linguistic differences between non-English languages. Lastly, formal testing of local item dependence (LID) was not conducted. Although the high discrimination parameters and observed item correlations suggest minimal local dependence, it is possible that some of the high discrimination estimates reflect item redundancy, which is common in brief screening instruments such as the K10.

## Conclusion

This study provides robust evidence supporting the psychometric soundness of the K10 in a South African context. It functions as a unidimensional, internally consistent, and discriminating measure of psychological distress. However, marginal reliability and information curves indicate it is most precise for detecting moderate-to-high distress levels, with potential for refinement in screening for milder symptoms. Additionally, the differential item functioning analysis revealed that while Items 4, 5, 7, 8, and 10 showed statistically significant differences between English-speaking and non-English–speaking respondents, the effect sizes were negligible, suggesting that language does not meaningfully bias responses. These findings support the continued use of the K10 across South Africa’s multilingual populations, though future research could further examine item performance across specific indigenous languages to ensure optimal cultural and linguistic applicability.

## Data Availability

The data used in this study are publicly available for research purposes through the Human Sciences Research Council (HSRC) data repository. The dataset can be accessed at: https://repository.hsrc.ac.za/.
